# Alleviating Pancreatic Cancer-Associated Pain Using Endoscopic Ultrasound-Guided Neurolysis

**DOI:** 10.3390/cancers10020050

**Published:** 2018-02-15

**Authors:** Kosuke Minaga, Mamoru Takenaka, Ken Kamata, Tomoe Yoshikawa, Atsushi Nakai, Shunsuke Omoto, Takeshi Miyata, Kentaro Yamao, Hajime Imai, Hiroki Sakamoto, Masayuki Kitano, Masatoshi Kudo

**Affiliations:** 1 Department of Gastroenterology and Hepatology, Kindai University Faculty of Medicine, Osaka-Sayama 589-8511, Japan; mamoxyo45@gmail.com (M.T.); ky11@leto.eonet.ne.jp (K.K.); t.yoshikawa113@gmail.com (T.Y.); nakai_agmc@yahoo.co.jp (A.N.); shunsuke.oomoto@gmail.com (S.O.); miyatchi77@yahoo.co.jp (T.M.); yamaken_volvo@yahoo.co.jp (K.Y.); codenamegenchan1023@gmail.com (H.I.); m-kudo@med.kindai.ac.jp (M.K.); 2 Department of Gastroenterology, Katsuragi Hospital, Kishiwada 596-0825, Japan; hiroki.sakamoto@nifty.com; 3 Second Department of Internal Medicine, Wakayama Medical University, Wakayama 641-8509, Japan; kitano@wakayama-med.ac.jp

**Keywords:** endoscopic ultrasound, EUS, EUS-guided neurolysis, neurolysis, interventional EUS, pancreatic cancer, pain

## Abstract

The most common symptom in patients with advanced pancreatic cancer is abdominal pain. This has traditionally been treated with nonsteroidal anti-inflammatory drugs and opioid analgesics. However, these treatments result in inadequate pain control or drug-related adverse effects in some patients. An alternative pain-relief modality is celiac plexus neurolysis, in which the celiac plexus is chemically ablated. This procedure was performed percutaneously or intraoperatively until 1996, when endoscopic ultrasound (EUS)-guided celiac plexus neurolysis was first described. In this transgastric anterior approach, a neurolytic agent is injected around the celiac trunk under EUS guidance. The procedure gained popularity as a minimally invasive approach and is currently widely used to treat pancreatic cancer-associated pain. We focus on two relatively new techniques of EUS-guided neurolysis: EUS-guided celiac ganglia neurolysis and EUS-guided broad plexus neurolysis, which have been developed to improve efficacy. Although the techniques are safe and effective in general, some serious adverse events including ischemic and infectious complications have been reported as the procedure has gained widespread popularity. We summarize reported clinical outcomes of EUS-guided neurolysis in pancreatic cancer (from the PubMed and Embase databases) with a goal of providing information useful in developing strategies for pancreatic cancer-associated pain alleviation.

## 1. Introduction

Pancreatic cancer has one of the worst prognoses among all solid carcinomas. The 5-year overall survival in pancreatic cancer remains dismal, with approximately 5–10% of patients surviving; more than half of the patients do not survive beyond 1 year [1,2]. Up to 80% of patients with pancreatic cancer experience abdominal and back pain, with 50–70% suffering from severe pain [3,4,5]. Because patients frequently present at an advanced stage, palliative care and not curative intent tends to be the primary goal. Pain control is a major goal of palliative care in advanced pancreatic cancer. Conventionally, pain is alleviated using nonsteroidal anti-inflammatory agents and/or opioid analgesics, following the three-step analgesic ladder pain management strategy recommended by the World Health Organization [6]. However, pain is difficult to control in some cases presenting a challenge to the physician. Further, some patients experience serious drug-related side effects that can markedly reduce quality of life. Under such circumstances, celiac plexus neurolysis (CPN), in which the celiac plexus (CP) is chemically ablated, has been widely performed as an alternative treatment for alleviating cancer-associated pain [4,7]. For several years, CPN had been performed percutaneously or during open surgery. Anterior or posterior percutaneous CPN can be performed under the guidance of transabdominal ultrasound, fluoroscopy, or computed tomography [7].

Endoscopic ultrasound-guided celiac plexus neurolysis (EUS-CPN) is a relatively new technique first described in 1996 [8]. In EUS-CPN, a neurolytic agent is injected around the celiac trunk using a linear-array echo endoscope. Since the time it was first described, EUS-CPN has been widely applied as a minimally invasive approach in treating pancreatic cancer-associated pain. The current National Comprehensive Cancer Network guidelines (version 3, 2017, National Comprehensive Cancer Network, Fort Washington, PA, USA) recommend EUS-CPN for treatment of severe cancer-associated pain [9]. Other EUS-guided techniques including EUS-guided celiac ganglia neurolysis (EUS-CGN) [10] and EUS-guided broad plexus neurolysis (EUS-BPN) [11] have recently been developed with a goal of improving the efficacy of this endoscopic technique. EUS-guided neurolysis is thought to be safer than the conventional percutaneous approach because EUS, particularly with color Doppler technology, provides detailed real-time imaging of blood vessels around the gastric lumen. However, as these EUS-guided techniques have gained widespread popularity, serious procedure-related adverse effects including ischemic and infectious complications have also been reported [12,13]. The aim of this review is to summarize clinical outcomes of EUS-guided neurolysis in pancreatic cancer with a goal of providing information useful for development of strategies to alleviate pancreatic cancer-associated pain. 

## 2. Literature Review Methodology

This review used electronic literature searches of the PubMed and Embase databases to identify articles focused on EUS-guided neurolysis published during the period from October 1996 to September 2017. Search terms used were “EUS OR endoscopic ultrasound” AND “neurolysis”. Our search was limited to articles published in the English language. Based on the title and abstract, we selected articles for full text review. In addition, bibliographies of the selected articles were manually searched to find additional relevant articles that were also reviewed in detail. Overall, we identified 50 references on EUS-guided neurolysis comprising 34 original articles [8,10,11,14,15,16,17,18,19,20,21,22,23,24,25,26,27,28,29,30,31,32,33,34,35,36,37,38,39,40,41,42,43,44], 11 case reports [45,46,47,48,49,50,51,52,53,54,55] and five systematic reviews [5,7,56,57,58].

## 3. Indications for EUS-Guided Neurolysis

EUS-guided neurolysis is mainly indicated in patients with chronic abdominal and back pain associated with upper gastrointestinal malignancies including pancreatic cancer. Patients with pancreatic cancer who are candidates for surgery with curative intent usually do not present with pain; on other hand, patients with pancreatic cancer at an unresectable stage who experience pain affecting their quality of life are good candidates for this treatment. Conventional treatment with analgesic drugs alleviates pain at least partially in most patients; however, some patients have inadequate pain control with this approach and some have drug-related side effects including dry mouth, constipation, nausea, vomiting and dependence [4,59]. In such cases, EUS-guided neurolysis is a useful alternative treatment that may reduce risk of drug-related side effects. Regarding timing of EUS-guided neurolysis, Wyse et al. reported that early EUS-CPN performed during diagnostic EUS provided better pain relief than conventional pain management and prevented progressive increases in morphine consumption [27]. Thus, EUS-guided neurolysis may be effective not only during follow-up but also at the time of initial cancer detection. To date, no randomized controlled trials comparing percutaneous and EUS-guided neurolysis have been conducted; therefore, the optimal initial approach remains unclear.

Contraindications to EUS-guided neurolysis include bleeding tendency (prothrombin time international normalized ratio >1.5, platelet count <50,000/µL) and cardiorespiratory instability prohibiting adequate sedation. Presence of esophageal or gastric varices may be a relative contraindication due to an increased risk of bleeding. Other relative contraindications include distorted or surgically altered anatomy, making it difficult to clearly visualize anatomic landmarks such as the celiac trunk or celiac ganglia under EUS guidance, direct tumor invasion or congenital anatomic malformations of the celiac or superior mesenteric artery [60].

## 4. Anatomy Relevant to Pancreatic Cancer Pain

It is speculated that abdominal pain associated with pancreatic cancer results from intra- and extra-pancreatic perineural invasion by cancer cells [3]. Complex neuronal pathways that transmit pain signals arise in the pancreas and travel to higher centers of the central nervous system through thoracic splanchnic nerves. Afferent neurons from the pancreas connect to the CP; electrical signals are then transmitted through dorsal root ganglia at the T12–L2 spinal level [61].

The CP is the largest plexus in the autonomic nervous system, composed of ganglia that surround the celiac trunk with sympathetic, parasympathetic and visceral sensory fibers and extending from the origin of the celiac artery (CA) to the origin of the superior mesenteric artery. The CP consists of right and left celiac ganglia which are located anterior to the aorta, slightly to the left and cephalad to the celiac trunk and medial to the left adrenal gland at the T12–L2 level [7,43,61]. The superior mesenteric plexus and inferior mesenteric plexus are situated on the lateral and anterior aspects of the aorta, respectively, between the origin of the superior mesenteric artery and the inferior mesenteric artery. The CP, superior mesenteric plexus and inferior mesenteric plexus consist of a network of both sympathetic and parasympathetic nerve fibers [43]. These plexuses are believed to play an indispensable role in pain perception in pancreatic cancer patients [62].

## 5. Endoscopic Procedures in EUS-Guided Neurolysis

### 5.1. Pretreatment Procedure

Hydration with intravenous saline solution (500–1000 mL) is recommended before the endoscopic procedure to minimize risk of hypotension. Patients are placed in the left lateral position under moderate sedation with various combinations of intravenous midazolam, propofol, and/or fentanyl. Vital signs are continuously monitored during the procedure with an automated noninvasive blood pressure device, electrocardiogram tracing and pulse oximetry. Before the endoscopic procedures, pain scores are evaluated objectively using a visual analog scale, a numeric rating scale, or a 10-point Likert pain score.

### 5.2. Endoscopic Procedure

#### 5.2.1. EUS-Guided Celiac Plexus Neurolysis (EUS-CPN) 

**Figure 1:**
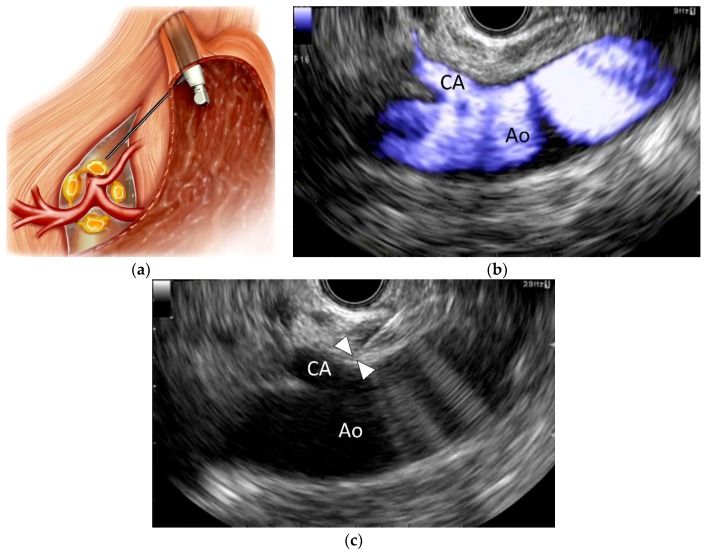
Endoscopic ultrasound-guided celiac plexus neurolysis (EUS-CPN). (**a**) Schematic of EUS-CPN; (**b**) Color flow EUS image from the lesser curvature of the stomach showing a longitudinal view of the aorta (Ao) and celiac artery (CA); (**c**) EUS image of EUS-CPN during needle puncture. A 22-gauge needle was advanced adjacent to the CA origin. Arrowheads indicate the needle tip. Blue: vascular flow.

EUS-CPN, first described in 1996 by Wiersema and Wiersema [8], is a relatively new technique in which a local anesthetic (bupivacaine or lidocaine) and a neurolytic agent (absolute alcohol or phenol) are injected around the CP under EUS guidance (Figure 1). EUS-CPN can be performed with either an oblique-viewing or forward-viewing curved linear-array echo endoscope [8,23,24]. Under moderate sedation, the echo endoscope is passed per-orally into the esophagus. Under endoscopic visualization, the echo endoscope is advanced through the gastroesophageal junction into the stomach. EUS imaging from the posterior lesser curvature of the gastric body allows visualization of the longitudinal view of the aorta. The aorta is traced distally to the origin of the CA, which is the first major branch below the diaphragm. The CP per se cannot be identified as a clear structure but is located based on its position around the celiac trunk. A 19- or 22-gauge aspiration needle filled with normal saline solution is prepared, passed through the biopsy channel and affixed to the hub. If a specially designed 20-gauge “spray needle” with multiple side holes is available [63], it could be used to spread the desired agent across a larger area.

EUS-CPN can be performed via a unilateral approach or a bilateral approach [19,56]. In the unilateral approach, the neurolytic agent is injected adjacent to a point just above the celiac trunk; in the bilateral approach, the agent is injected on both sides of the celiac trunk. For the unilateral approach, the needle is inserted under EUS guidance adjacent to the CA origin. To avoid transient pain induced by chemical stimulation with a neurolytic agent, 2–3 mL of a local anesthetic (bupivacaine or lidocaine) is initially injected. Then, a mixed solution of absolute alcohol and contrast medium is injected around the celiac trunk. The total volume of alcohol injected is usually 10–20 mL in EUS-CPN. For the bilateral approach, the probe is rotated clockwise toward the patient’s left at the level of the CA until the celiac trunk is no longer visualized but the aorta is still visible. The agent is injected in this region. Subsequently, the same process is carried out on the opposite side of the aorta (with counter-clockwise rotation).

To learn the procedure of Hands-on training using an animal model may be helpful in learning the EUS-guided neurolysis procedure. Bhutani et al. developed a swine model for teaching EUS and successfully performed EUS-CPN using the model. They concluded that the swine model was useful for hands-on training in EUS-guided interventions [14].

#### 5.2.2. EUS-Guided Celiac Ganglia Neurolysis (EUS-CGN) 

**Figure 2:**
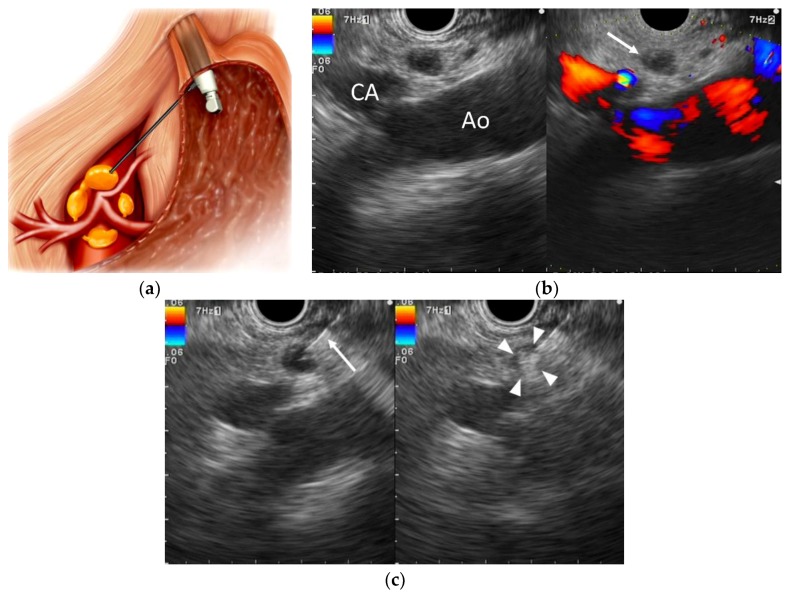
Endoscopic ultrasound-guided celiac ganglia neurolysis (EUS-CGN). (**a**) Schematic of EUS-CGN; (**b**) EUS image from the lesser curvature of the stomach showing the celiac ganglion located anterior to the aorta (arrow). Ao: aorta, CA: celiac artery. (**c**) EUS image of EUS-CGN before and after injection of a neurolytic agent. The ganglion has a hyperechoic appearance (arrowheads). Blue: vascular flow away from the transducer; Red: vascular flow towards the transducer.

In EUS-CGN, first described by Levy et al. [10], a neurolytic agent is directly injected into celiac ganglia (Figure 2). Several studies demonstrated that EUS could visualize celiac ganglia in 62.5–89.4% of patients [25,44,64,65]. After visualization of the celiac trunk, the scope is rotated clockwise, enabling visualization of the left adrenal gland. Most frequently, the celiac ganglia can be visualized on the left of the CA between the aorta and the left adrenal gland, at a level between the CA and the left renal artery. In some cases, celiac ganglia can be visualized cephalad to the CA. Under EUS guidance, hypoechoic round or nodular structures connected by hypoechoic thread-like structures in the periphery of this region are defined as celiac ganglia. Celiac ganglia vary in number (1 to 5), size (diameter 0.5–4.5 cm) and location (T12–L2) [66]. In EUS-CGN, each ganglion is punctured with a 19- or 22-gauge aspiration needle and absolute alcohol is injected until the entire ganglion becomes hyperechoic, reflecting alcohol injection. A volume of 1–2 mL alcohol is injected in each ganglion. An effort is made to puncture as many visualized ganglia as possible, to maximize efficacy.

#### 5.2.3. EUS-Guided Broad Plexus Neurolysis (EUS-BPN) 

**Figure 3:**
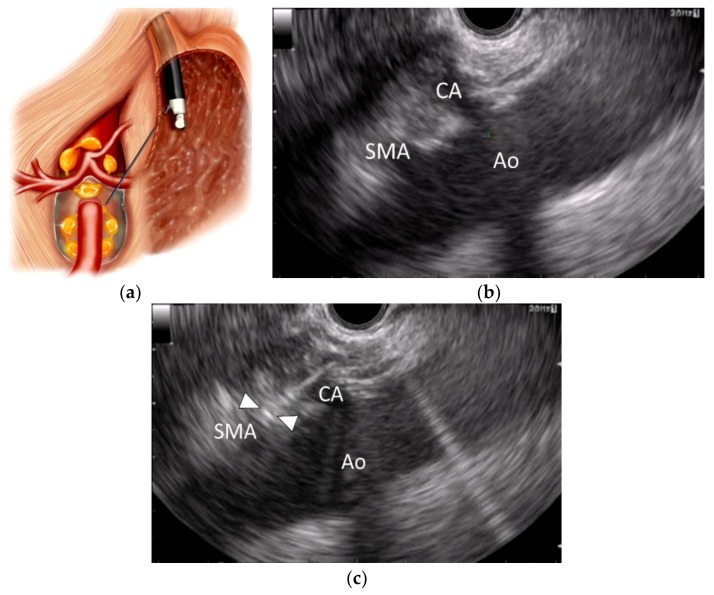
Endoscopic ultrasound-guided broad plexus neurolysis (EUS-BPN). (**a**) Schematic of EUS-BPN; (**b**) EUS image from the lesser curvature of the stomach showing a longitudinal view of the aorta (Ao), celiac artery (CA) and superior mesenteric artery (SMA); (**c**) EUS image of EUS-BPN during needle puncture. A 25-gauge needle was advanced adjacent to the SMA. Arrowheads indicate the needle tip.

EUS-BPN is a recently developed variation of EUS-guided neurolysis, first described in 2010 by Sakamoto et al. [11]. In EUS-BPN, a neurolytic agent is injected around the origin of the superior mesenteric artery to produce a wider distribution of neurolytic agent (Figure 3). In EUS-BPN, the probe is rotated clockwise toward the patient’s left at the level of the superior mesenteric artery until the origin of the superior mesenteric artery can no longer be visualized but the aorta is still visible. Because the aspiration needle is advanced deeper in EUS-BPN than in EUS-CPN, use of a 25-gauge needle is preferable to provide safety and flexibility during needle advancement into the target area. A 25-gauge aspiration needle filled with normal saline solution is prepared and introduced through the biopsy channel. Under EUS guidance, the needle is advanced adjacent and anterior to the lateral aspect of the aorta at a level above or next to the superior mesenteric artery. Two or 3 mL of a lidocaine solution is injected to prevent transient pain caused because of neurolytic agent injection. Subsequently, a neurolytic agent (absolute alcohol) is injected up to a maximum volume of 10 mL. Next, the process is repeated on the opposite side of the aorta (with counter-clockwise rotation), if possible.

## 6. Efficacy of EUS-Guided Neurolysis

### 6.1. EUS-CPN

In an initial report of EUS-CPN use, 30 patients with intra-abdominal malignancy-associated pain (with 25 pancreatic cancer patients) underwent EUS-CPN. Pain improvement was achieved at 2, 4, 8 and 12 weeks after EUS-CPN in 79–88% of the patients [8]. Several clinical trials of EUS-CPN have been published since the first report [15,16,17,18,19,22,26,27,28,29,30,33,34,35,36,37,38,39,40,42] (Table 1). Two meta-analyses of the utility of EUS-CPN in unresectable abdominal cancer-associated pain showed an alleviation rate of 73–80% with treatment duration of approximately 1–2 months [57,58]. According to a recent systematic review by Nagels et al., EUS-CPN should be considered in pancreatic cancer patients whose pain is inadequately controlled with systemic analgesics or who suffer from significant drug-related side effects [7]. To date, there has been only one randomized controlled trial which assessed EUS-CPN in comparison with conventional drug-based pain management [27]. According to the trial report by Wyze et al. 96 patients with advanced pancreatic cancer were randomly assigned to early EUS-CPN (i.e., EUS-CPN was performed during diagnosis of pancreatic cancer) or conventional drug-based pain management; early CPN was found to be superior in pain relief at three months compared with conventional pain management [27]. A Cochrane Review of six studies (358 patients) showed that in comparison with control, EUS-CPN afforded pain relief at four and eight weeks (visual analog score −0.42 (−0.70 to −0.13) and −0.44 (−0.89 to −0.01), respectively) and that it was associated with significant reduction in post-procedural analgesic consumption (*p* < 0.00001) [5]. These results indicate that EUS-CPN may be superior to drug-based management for pain relief in advanced pancreatic cancer patients.

Differences between the two major approaches of EUS-CPN were evaluated by LeBlanc et al. in a randomized study comprising 50 pancreatic cancer patients comparing efficacy of unilateral and bilateral CPN; pain relief was reported in 69% patients who underwent unilateral injection and in 81% patients who underwent bilateral injection, with no statistically significant differences [28]. Sahai et al. evaluated efficacy of the two approaches in 160 patients and found that bilateral CPN was the only determinant of >50% pain relief by day seven [19]. The most recent meta-analysis comparing the two approaches, by Lu et al. included six studies (437 patients); no significant difference was found between the approaches in short-term pain relief or response to treatment. However, EUS-guided bilateral CPN was associated with significantly lesser analgesic consumption than unilateral CPN [56].

Another new technique is EUS-guided ethanol tumor ablation combined with CPN. A recent study by Facciorusso et al. compared the efficacy and safety of EUS-guided ethanol tumor ablation combined with CPN (*n* = 65) with those of CPN alone (*n* = 58) for pain management in advanced pancreatic cancer patients (*n* = 123). The study found that EUS-guided tumor ablation combined with CPN appeared to be superior to CPN alone with respect to pain relief and overall survival [42,53].

There is insufficient evidence to evaluate the impact of EUS-CPN on overall survival in pancreatic cancer. A retrospective case–control study of 417 patients by Fujii-Lau et al. suggested that celiac neurolysis (including EUS-CPN and EUS-CGN) was an independent determinant of shortened survival in pancreatic cancer [40]. According to a meta-analysis by Yan et al. comprising five randomized controlled trials on the effect of non-EUS-guided CPN in pain management in advanced pancreatic cancer, CPN use was associated with a significant reduction in pain intensity and analgesic consumption; however, CPN did not affect survival [67]. In contrast, in a study by Fujii-Lau, EUS-guided neurolysis was associated with longer survival than non-EUS-guided approaches [40]. Further prospective studies are needed to evaluate impact of EUS-guided neurolysis on patient survival.

### 6.2. EUS-CGN

As previously described, EUS allows visualization of celiac ganglia in 62.5–89.4% of patients [25,44,64,65]. Kappelle et al. reported that a total of 204 ganglia in 83 patients were detected during 97 consecutive EUS procedures and that the mean length of the major axis of the ganglia was 8.1 mm [44]. The ganglia were visualized anterior to the aorta and/or to the left of the CA in 94% of patients [44]. A retrospective study by Ascunce et al. suggested that visualization of celiac ganglia with direct CGN was the best determinant of pain-relief response following EUS-guided celiac neurolysis [25]. In a randomized multicenter trial by Doi et al. EUS-CGN was more effective than EUS-CPN in providing pain relief (pain-relief response of 73.5% vs. 45.5%, respectively, *p* = 0.02) [35]. Considering these findings, EUS-CGN may be more effective than EUS-CPN for pain relief in advanced pancreatic cancer. Most recently, Kappelle et al. successfully visualized the area of alcohol spread following various EUS-guided neurolysis approaches and alcohol doses in a human cadaver model [44]. In their study, EUS-CGN was performed with 1 mL (low volume) or 4 mL (high volume) alcohol injection per ganglion. Neurolytic-spread area was assessed by visualizing spread of an orange dye mixed with the alcohol. After low-volume EUS-CGN in cadavers, the neurolytic agent spread well beyond the targeted ganglion. High-volume EUS-CGN resulted in wider ethanol spread, also reaching undefined ganglia. The authors concluded that high-volume EUS-CGN is preferable to low-volume EUS-CGN because it is likely to achieve more thorough neurolysis [44].

In a pilot study by Wang et al. EUS-guided implantation of iodine-125 (^125^I) around the celiac ganglia was performed in 23 advanced pancreatic cancer patients. The authors found that EUS-guided celiac ganglia irradiation with ^125^I seeds was effective for pain relief and reduced analgesic consumption at two weeks following the procedure, with no major procedure-related complications [32].

### 6.3. EUS-BPN

An initial retrospective study by Sakamoto et al. compared efficacy and safety of EUS-CPN and EUS-BPN in pancreatic cancer pain management. The results of the study suggested that EUS-BPN was more effective, especially in patients with extensive spread of cancer within the abdominal cavity beyond the distribution of the CP and that the procedure did not result in serious complications [11]. In several studies, EUS-CPN, EUS-CGN and EUS-BPN have shown satisfactory results and excellent safety profiles, indicating that they are all promising methods; however, the efficacy of these techniques is not assured. Therefore, we conducted a study to explore determinants of pain-relief response in 112 patients undergoing EUS-guided neurolysis for pancreatic cancer-associated abdominal pain. Multivariable analysis revealed that EUS-BPN in combination with EUS-CGN was a significant determinant of pain-relief response [43]. In our study, the neurolytic-spread area was divided into six sections and assessed using post-procedural computed tomography. The number of sections with neurolytic spread was higher in patients who underwent EUS-BPN in combination with EUS-CGN than in patients who underwent EUS-BPN alone. This finding suggests that wider distribution of neurolytic agent may be associated with better pain relief. Because EUS-BPN has been reported only at a single institution currently, a multicenter study with a larger number of patients is required to confirm efficacy and safety of this technique.

## 7. Complications of EUS-Guided Neurolysis

Although EUS-guided neurolysis has been shown to be a safe procedure, side effects and complications can occur during and after the procedure. A recent review on interventional EUS-related safety and complications comprising 15 studies found that complications occurred in 21% of 661 patients [68]. Most of the reported complications were minor and self-limiting, usually lasting less than two days and were attributed to disruption of sympathetic activity [20]. According to a systematic review by Nagels et al. frequent complications related to EUS-CPN were diarrhea (18%) and hypotension (20%) resulting from sympatholytic reactions [7]. A transient increase in pain occurred in 1.5–8% of patients after EUS-CPN [7]. Signs of alcohol intoxication resulting from the procedure were reported only in Japan [11].

Serious complications have been reported to be uncommon, occurring in only 0.2% of EUS-guided neurolysis cases [68]. Table 2 shows all major complications reported following EUS-guided neurolysis in pancreatic cancer patients [46,48,49,50,51,52,54,55]. Among these, ischemic complications, which can be fatal, are considered the most serious adverse events. Four cases of acute paraplegia have been reported; in all four cases, the paraplegia was permanent [48,49,52,55]. Paraplegia following EUS-guided neurolysis is thought to be caused by acute spinal cord ischemia resulting from injury to the anterior radicular artery (artery of Adamkiewicz) or from vasospasm associated with neurolytic agent injection. A recent case report first described acute respiratory failure resulting from bilateral diaphragmatic paralysis following EUS-CPN [54]. In that case, paralysis involved cranial spread of neurolytic agent from the CP toward the diaphragm; the neurolytic agent made contact with both phrenic nerves which innervate the diaphragm from below. Hepatic and splenic infarction and bowel ischemia occurred in two patients, both of whom died due to multiorgan failure and sepsis [50,51]. Possible mechanisms of injury include diffusion of neurolytic agent adjacent to the CA resulting in arterial vasospasm reflecting the sclerosing effect of absolute ethanol and arterial embolization following injection of neurolytic agent. Because serious and even fatal complications can occur, endosonographers should bear the risk of ischemic complications in mind when considering EUS-guided neurolysis and all patients should be informed about these serious complications before the procedure.

## 8. Determinants of Pain-Relief Response

Several studies have investigated determinants of pain-relief response following EUS-guided neurolysis. Several studies have reported that a wider distribution of neurolytic agent is associated with better pain-relief response. In a retrospective study by Iwata et al. including 47 patients who underwent EUS-CPN, multivariable analysis revealed that direct tumor invasion of the celiac axis and distribution of alcohol on only the left side of the CA were significant factors associated with negative pain-relief response to EUS-CPN [26]. Our retrospective study of 112 patients with advanced pancreatic cancer who underwent EUS-guided neurolysis showed that EUS-BPN in combination with EUS-CGN (combination method) was a significant predictor of good pain-relief response. The results of our study also showed that the number of neurolytic-spread areas in post-procedural CT was significantly higher in patients who received the combination method than in those treated with EUS-BPN alone. This result suggests that larger spread of neurolytic agent might contribute to improved efficacy of the combination method.

Most recently, Bang et al. prospectively analyzed data from 51 patients who underwent EUS-CPN for abdominal pain caused by advanced pancreatic cancer to examine whether a correlation existed between increased heart rate and treatment outcomes. The authors found that heart rate change (increase of ≥15 beats/min for 30 s) during alcohol injection was associated with improved pain-relief response and quality of life [41].

One explanation for the reduction in EUS-CPN pain improvement after 2–3 months following the procedure is that the neurolytic agent does not remain in the targeted anatomic location but flows away from the injection site because of its high fluidity [21]. This suggests that neurolytic agent delivery in a solid or gel form may result in enhanced efficacy and safety. A study by Obstein et al. described the use of EUS-CPN with a reverse-phase polymer in a porcine model. The study found that formation of a gel plug at the exact location of the celiac ganglia prevented diffusion of the injected agent and prolonged the duration of analgesic effect [21].

## 9. Conclusions and Future Directions

EUS-guided neurolysis has been increasingly used as minimally invasive intervention for pain relief in patients with advanced pancreatic cancer. Recent systematic reviews on the procedure have reported an efficacy of approximately 80% with few serious complications. Three different neurolytic approaches exist, comprising EUS-CPN, EUS-CGN and EUS-BPN. A bilateral approach in EUS-CPN is associated with lower analgesic consumption although efficacy of bilateral and unilateral EUS-CPN appears similar. EUS-CGN may be more effective than unilateral EUS-CPN without an increase in complications. EUS-BPN in combination with EUS-CGN may provide better pain relief than either approach alone, although the combination approach may be technically challenging. In several small studies, EUS-CPN, EUS-CGN and EUS-BPN have been reported to show satisfactory results and excellent safety profiles; however, efficacy of these techniques is not assured. Moreover, no studies comparing conventional percutaneous and EUS-guided neurolysis can be found. Drug-based pain management has improved with recent development of new analgesic agents. Future prospective, well-designed studies comparing the CPN techniques and analgesic pain management using new drugs are essential to establish the role of EUS-guided neurolysis as a pain-management modality in pancreatic cancer. Comparison with other interventional procedures including radiotherapy and intrathecal therapy may also be warranted. Further, to achieve lasting pain relief, neurolytic agents and the associated delivery methods may need improvement. As the use of EUS-guided neurolysis has become widespread, serious adverse events including ischemic and infectious complications have been described increasingly frequently. Endosonographers should bear the possibility of serious complications in mind when considering EUS-guided neurolysis.

## Figures and Tables

**Table 1:** Clinical studies of efficacy and safety of endoscopic ultrasound (EUS)-guided neurolysis.

First Author (Year) [Reference]	Study Design	No. of Patients	Procedure	Outcomes	Complications
Wiersema (1996) [8]	Prospective? Non-randomized	30	EUS-CPN Bilateral	Pain improvement in 79 to 88% of patients with a median follow-up of 10 weeks	Self-limited complications Diarrhea 13.3% Pain increase 3.3%
Gunaratnam (2001) [15]	Prospective Non-randomized	58	EUS-CPN Bilateral	Decline in pain score after EUS-CPN in 78% of patients	No major complications Pain increase 8.6%
Tran (2006) [16]	Retrospective Non-randomized	8	EUS-CPN Unilateral	Pain improvement in 70% of 10 procedures (8 patients)	Not described
Sakamoto (2006) [17]	Retrospective Non-randomized	13	EUS-CPN Bilateral	Pain improvement in 84.6% of patients	Self-limited complications Inebriation 7.7% Pain increase 7.7% Hypotension 15.4%
Levy (2008) [10]	Retrospective Non-randomized	36 (Malignant 18)	EUS-CGN	Pain improvement in 94% of patients	Pain increase 36.1% Hypotension 33.3% Diarrhea 16.6%
Ramirez-Luna (2008) [18]	Retrospective Non-randomized	11	EUS-CPN Unilateral	Pain improvement in 72% of patients at 4 weeks after CPN	No major complications Transient pain increase 45.4%
Sahai (2009) [19]	Retrospective Non-randomized	160 (Malignant 81)	EUS-CPN Bilateral 89 Unilateral 71	Pain improvement; 70.4% (bilateral) vs 45.9% (unilateral) Bilateral CPN is more effective than unilateral CPN	Retroperitoneal bleeding 1% (bilateral CPN)
Sakamoto (2010) [11]	Retrospective Non-randomized	67	EUS-CPN 34 EUS-BPN 33	Reduction in pain score on days 7 and 30; EUS-BPN > EUS-CPN	No serious complications No cases of prolonged hospitalization
Ascunce (2011) [25]	Retrospective Non-randomized	64	EUS-CGN 40 EUS-CPN 24	Pain improvement at 1 week after neurolysis; 65.0% (CGN) vs. 25.0% (bilateral CPN)	Transient pain increase 1.6%, Diarrhea 23.4%, Hypotension 1.6%
Iwata (2011) [26]	Retrospective Non-randomized	47	EUS-CPN Unilateral	Pain improvement; 68.1% Complete pain relief; 36.2%	Transient hypotension 17.0%, Inebriation 8.5%, Diarrhea 23.4%
Wyse (2011) [27]	Prospective Randomized	48	EUS-CPN Bilateral	Randomized trial; EUS-CPN vs conventional drug-based pain management Pain relief at 3 months; CPN > drug-based pain management	No evidence of early or late complications
LeBlanc (2011) [28]	Prospective Randomized	50	EUS-CPN Bilateral 21 Unilateral 29	Randomized trial; bilateral CPN vs unilateral CPN Pain relief and survival; no difference between the groups	Transient pain increase 36%, Hypotension 2%
Wiechowska-Kozłowska (2012) [29]	Retrospective Non-randomized	29	EUS-CPN Bilateral	Pain improvement; 86% Complete pain relief; 14%	Transient diarrhea 10.3%, Hypotension 3.4%, Pain increase 6.9%
Wang (2012) [32]	Prospective Non-randomized	23	EUS-guided irradiation	EUS-guided celiac ganglion irradiation (iodine-125 seeds) Pain improvement in 82.6% of patients at 2 weeks	No major complications Constipation 21.7% Nausea 8.7%
Leblanc (2013) [33]	Prospective Randomized	20	EUS-CPN Unilateral (+EUS-CGN)	Randomized trial; EUS-CPN using 10 mL vs. 20 mL alcohol Similar clinical outcomes between the groups	Self-limited complications Lightheadedness 5% Diarrhea 10% Nausea 15%
Seicean (2013) [34]	Retrospective Non-randomized	32	EUS-CPN Unilateral	Pain improvement in 75% of patients	No complications
Doi (2013) [35]	Prospective Randomized	68	EUS-CGN 34 EUS-CPN 34	Randomized trial; EUS-CGN vs. EUS-CPN (unilateral) Pain improvement; 73.5% (CGN) vs. 45.5% (CPN) Complete pain relief; 50% (CGN) vs. 18.2% (CPN)	Transient hypotension 4.5%, Inebriation 3.0%, Pain increase 25.4%, Diarrhea 7.5%
Téllez-Ávila (2013) [37]	Retrospective Non-randomized	53	EUS-CPN Unilateral 21 Bilateral 32	Bilateral vs. unilateral CPN No significant difference between the groups	No major complications Transient pain increase 1.9%
Si-Jie (2014) [36]	Retrospective Non-randomized	41	EUS-CGN 26 EUS-CPN 15	Pain improvement in 90.2% and 61.0% of patients at 1 week and at 3 months, respectively	Transient hypotension 4.9%
Ishiwatari (2014) [38]	Retrospective Non-randomized	22	EUS-CPN Phenol 6 Ethanol 16	Pain improvement in 83% and 69% of patients in the phenol and ethanol groups, respectively	Minor complications Phenol group 16.7%, ethanol group 37.5% Inebriation 12.5% (ethanol group)
Ishiwatari (2015) [39]	Prospective Non-randomized	9	EUS-CPN Phenol-glycerol	Complete, partial and no pain relief in 44.4%, 44.4% and 11.1% of patients at 7 days after the procedure	Minor complications 33.3%
Fujii-Lau (2015) [40]	Retrospective Non-randomized	230	EUS-CPN or EUS-CGN	EUS-guided celiac neurolysis was associated with longer survival compared with non-EUS approaches	Mild adverse events; 7 patients (1.7%) Moderate to severe adverse events; 5 patients (1.2%)
Bang (2016) [41]	Prospective Non-randomized	51	EUS-CPN Unilateral	Heart rate change during CPN in 49.0% of patients Better pain relief in the heart rate change cohort	Diarrhea 33.3%
Minaga (2016) [43]	Retrospective Non-randomized	112	EUS-BPN 65 EUS-BPN + EUS-CGN 47	Pain improvement in 78% of patient at 1 week EUS-BPN in combination with EUS-CGN is a predictor of a good pain response	Major; Paraplegia 1% Minor; Inebriation 8.0%, Hypotension 4.5%, Pain increase 3.6%, Diarrhea 3.6%
Facciorusso (2017) [42]	Retrospective Non-randomized	123	EUS-CPN 58 EUS-CPN + ablation 65	EUS-guided tumor ethanol ablation combined with EUS-CPN increased pain relief and complete pain response rate	No severe treatment-related complications

CPN, celiac plexus neurolysis; CGN, celiac ganglia neurolysis; BPN, broad plexus neurolysis.

**Table 2:** Major complications of EUS-guided neurolysis in pancreatic cancer.

First Author (Year) [Reference]	Complications	Procedure	Neurolytic Agents/Anesthetic Agents	Outcomes
Muscatiello (2006) [46]	Retroperitoneal abscess	CPN	Alcohol/Bupivacaine	EUS-guided puncture, complete resolution
Mittal (2012) [48]	Paraplegia	CGN + CPN	Alcohol/Bupivacaine	No improvement
Fujii-Lau (2012) [49]	Paraplegia	CGN + CPN	Alcohol/Bupivacaine	No improvement
Gimeno-García (2012) [50]	Celiac artery thrombosis, hepatic, kidney, splenic infarction, bowel ischemia	CPN Bilateral	Alcohol/Bupivacaine	Conservative treatment, died 8 days later
Jang (2013) [51]	Hepatic, splenic infarction, bowel ischemia	CPN Unilateral	Alcohol, triamcinolone acetonide/Bupivacaine	Conservative treatment, died 27 days later
Minaga (2016) [52]	Paraplegia	CPN Bilateral	Alcohol/Lidocaine	No improvement
Mulhall (2016) [54]	Bilateral diaphragmatic paralysis	CPN	No description	Mechanical ventilation, no improvement
Köker (2017) [55]	Paraplegia	CPN Bilateral	Alcohol/Bupivacaine	No improvement
